# Autotransplantation of Impacted Third Molars to DCIA Free Flap in Adolescent Patient: A Case Report

**DOI:** 10.3390/children12030370

**Published:** 2025-03-16

**Authors:** Benjamin Walch, Alexander Gaggl, Katharina Zeman-Kuhnert, Christian Brandtner

**Affiliations:** Department of Oral and Maxillofacial Surgery, Medical University Salzburg, 5020 Salzburg, Austria; a.gaggl@salk.at (A.G.); c.brandtner@salk.at (C.B.)

**Keywords:** microsurgical free flap, reconstructive surgical procedures, autotransplant, oral surgery, maxillofacial surgery, pediatrics, dentistry, pediatric, impacted teeth, ossifying fibroma, computer-aided design, computer-aided manufacturing

## Abstract

Introduction: Tooth autotransplantation is a well-established dental surgical procedure. However, third molar autotransplantation to bony free flaps is rarely performed. We present a case of two impacted wisdom teeth that were transplanted to a DCIA free flap using 3D printing technologies. Case report: A 10-year-old girl was diagnosed with ossifying fibroma. She underwent a segmental mandibular resection with nerve preservation and reconstruction using a DCIA free flap. Six years later, due to edentulism, wisdom tooth autotransplantation was performed with digital planning, thermoplastic vacuum-formed guides, and 3D-printed replicas. Postoperatively, splint fixation was required for 12 weeks due to mobility, and a minor wound complication resolved spontaneously. At the one-year follow-up, the transplanted teeth integrated successfully without resorption or ankylosis. Orthodontic treatment was initiated to optimize alignment. Conclusions: This case of an impacted third molar autotransplantation to a DCIA free flap in an adolescent patient after a non-malignant mandibular tumor resection and reconstruction demonstrates promising results. The application of 3D printing technology significantly enhances the feasibility of dental transplantation in challenging cases, particularly for suboptimal donor teeth such as impacted wisdom teeth, by enabling precise surgical planning and optimized recipient site preparation while also reducing damage to the grafted teeth during transplantation. Further research is needed to assess the role of tooth autotransplantation in bony free flaps.

## 1. Introduction

The transplantation of teeth into an edentulous site is a well-established dental surgical procedure with high long-term survival rates of about 90% [1,2,3]. This technique involves relocating healthy, sometimes impacted or extracted, teeth to replace missing ones. Although tooth autotransplantation was first introduced in the 1950s, it has gained renewed attention in recent years due to advancements in surgical techniques, diagnostic imaging, 3D printing, and a better understanding of the healing process [4,5].

Autotransplantation of teeth offers several advantages, particularly in young patients with developing jawbones. This procedure facilitates the optimal integration of a transplanted tooth while allowing for continued root growth and physiological adaptation. In contrast, dental implants present prosthetic challenges in adolescent patients, as ongoing jaw growth can lead to infraocclusion over time, resulting in the suboptimal positioning of the implant within the dentition [3,6,7,8].

However, the success of tooth autotransplantation depends on multiple factors, including the developmental stage of the donor tooth, the condition of the recipient site, the patient’s overall health and age, and the surgical technique employed. Clinicians must carefully evaluate these variables before considering autotransplantation as a viable treatment option, as potential complications such as root resorption, ankylosis, or the failure of the transplanted tooth to integrate with the surrounding bone may occur. Wisdom tooth autotransplantation presents additional challenges; however, advancements in technology may improve success rates by enhancing surgical precision and treatment planning. A successful new approach involves the use of 3D printing and CAD/CAM-guided techniques [9]. The 3D printing of model teeth and surgical guides has been shown to significantly reduce the preparation time for the alveolar socket and minimize the extra-alveolar time of the donor tooth. Additionally, it decreases the number of positioning trials required during transplantation, thereby reducing procedural trauma and improving clinical outcomes. As a result, it may enhance the survival rate of transplanted teeth while decreasing the risk of complications such as pulp necrosis, ankylosis, external root resorption, and impaired healing [10,11,12].

Despite its potential, tooth autotransplantation in bony free flaps remains an underexplored treatment modality, and there is a need for further case studies and long-term follow-up data to evaluate its long-term success rates, particularly in terms of functional outcomes and survival. Specifically, the efficiency of this technique concerning impacted third molars or situations where the donor tooth is not in optimal condition has not been fully explored yet.

This case report aims to provide a detailed account of a clinical instance of impacted wisdom tooth autotransplantation to a DCIA free flap, outlining the pre-surgical assessment, surgical procedure, postoperative care, and outcomes observed. The report will also highlight the challenges faced during the transplantation process, including the selection of the donor tooth, the management of the recipient site, and the post-operative complications. By sharing this case, we hope to contribute valuable insights to the growing body of literature on the clinical application of wisdom tooth autotransplantation and its potential as a viable treatment option in mandible free flap reconstruction of pediatric patients, especially when applying 3D printing technologies.

## 2. Case Report

A 10-year-old girl with a painless swelling in the left posterior mandible, initially noticed by her parents one month prior, was evaluated following a referral from her dentist. The swelling had gradually increased in size without associated pain, sensory disturbances, or signs of infection. The patient had no history of trauma, systemic illness, or relevant medical conditions. Clinical examination revealed mild facial asymmetry due to a firm, non-tender swelling at the posterior left mandibular body. Intraorally, buccal and lingual cortical expansion was observed, but the overlying mucosa was intact without ulceration or signs of infection. The sensory function of the inferior alveolar nerve was preserved.

**Figure 1:**
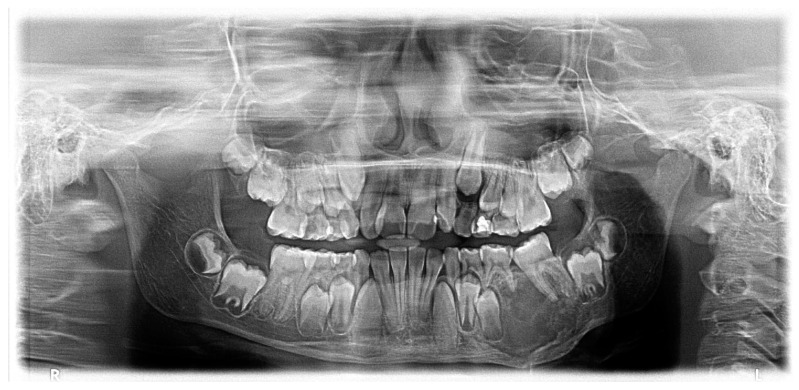
Initial OPG before resection showing a unilocular radiolucent lesion in the posterior left mandible.

Radiographic assessment using a panoramic radiograph (OPG) and cone-beam computed tomography (CBCT) revealed a well-defined, unilocular radiolucent lesion with a mixed radiopaque component. The lesion exhibited significant cortical expansion with thinning but without perforation. There was displacement of the teeth 36, 75, 74. However, root resorption was not observed. Based on these findings, ossifying fibroma was suspected. A tissue biopsy was performed under general anesthesia, and histopathological analysis confirmed the diagnosis, showing a well-demarcated lesion with a fibrocellular stroma interspersed with trabecular and woven bone (Figure 1).

**Figure 2:**
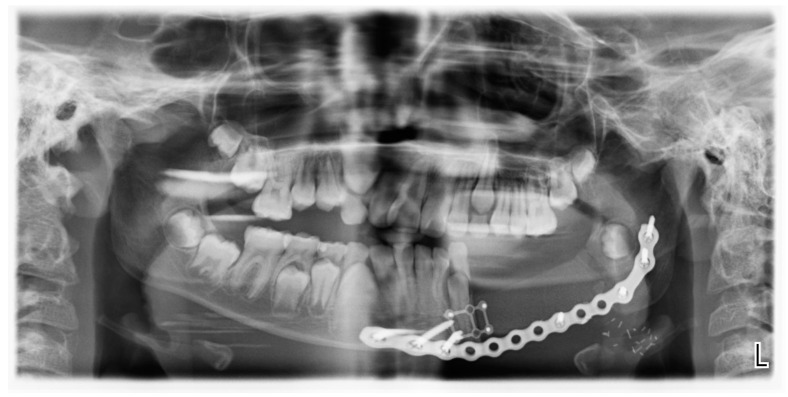
Postoperative OPG after segmental resection and DCIA free flap reconstruction.

**Figure 3:**
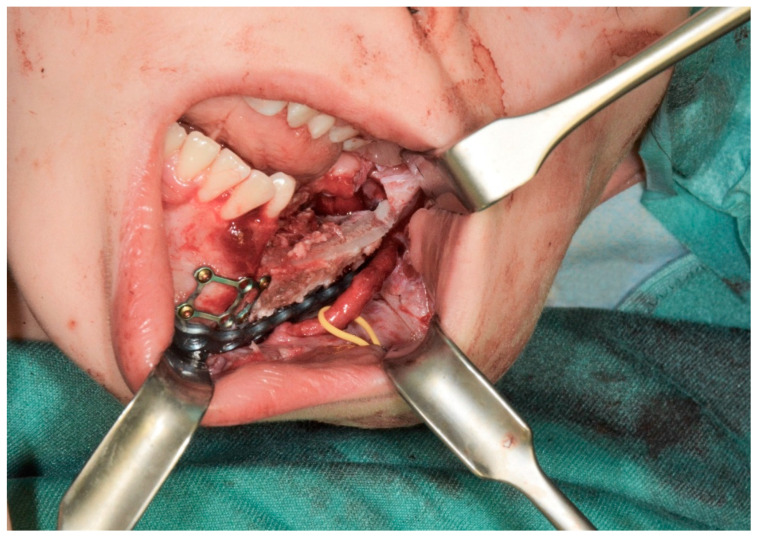
Intraoperative view after DCIA free flap reconstruction and lateralization of the inferior alveolar nerve.

Three months after the initial diagnosis, the patient underwent segmental mandibular resection with nerve lateralization and immediate reconstruction using a deep circumflex iliac artery (DCIA) flap. An intraoral approach provided access to the affected mandible, allowing for a segmental resection with clear margins. The inferior alveolar nerve was carefully preserved and repositioned to maintain sensory function. For reconstruction, a vascularized iliac crest bone flap was harvested, contoured to match the mandibular defect, and secured with titanium plates (Figure 2 and Figure 3). Next, microvascular anastomosis was performed to the facial artery and vein using a small submandibular incision.

The postoperative course was uneventful, with the patient experiencing minimal discomfort. The sensory function of the inferior alveolar nerve remained intact, and there were no signs of infection or graft failure. Follow-up at six and twelve months demonstrated a successful integration of the graft, stable occlusion, and no radiographic evidence of recurrence. Given the patient’s young age and ongoing craniofacial development, long-term monitoring was planned to assess skeletal growth and evaluate the potential need for secondary corrective procedures. Although a slight asymmetry in the facial contour was noted after reconstruction, no evidence of midline deviation, crossbite, or other signs of severe skeletal malocclusion was observed during the eight years of follow-up.

**Figure 4:**
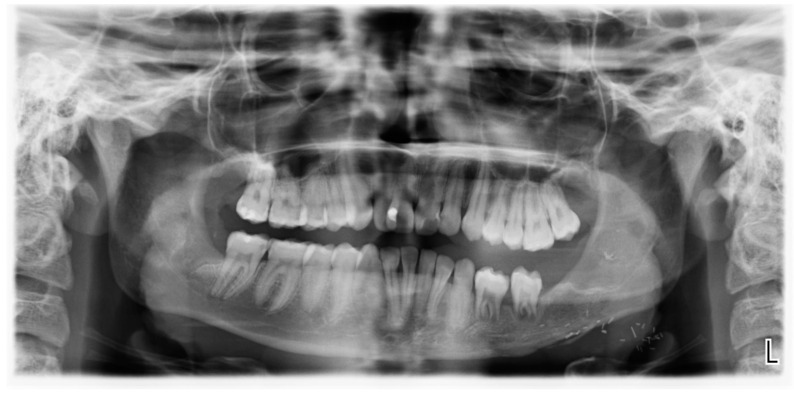
OPG after TTS removal.

Despite the edentulous state of the posterior left mandible, mandibular growth progressed regularly without the development of consecutive malocclusion. However, as skeletal maturity had not yet been fully reached, the patient expressed the need for dental rehabilitation six years later. Given the absence of dentition in the transplanted DCIA free flap, the indication for wisdom tooth autotransplantation was established. The left and right impacted mandibular third molars were selected as donor teeth and transplanted into the edentulous region. They were secured using flexible dental splinting with a titanium trauma splint (TTS) (Figure 4).

**Figure 5:**
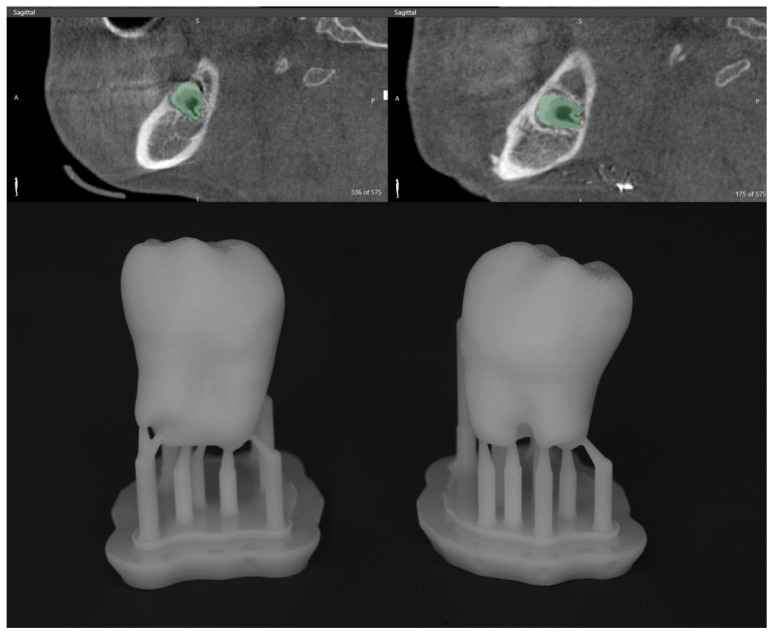
DICOM data segmentation and printed teeth replicas.

For precise surgical planning, the digital imaging and communications in medicine (DICOM) data from CBCT scans were exported from our clinical imaging and documentation software, DeepUnity Diagnost (Dedalus Healthcare Group, Version 1.1.1.1, Milan, Lombardy, Italy). Three-dimensional standard tessellation language (STL) models were generated using DICOM to PRINT software (3D Systems, Version 1.0.2.2055, Rock Hill, SC, USA), allowing for virtual preoperative planning. The mandible and wisdom teeth were subsequently 3D printed using a Formlabs 3BL printer (Formlabs, Somerville, MA, USA) (Figure 5).

Custom surgical guides were fabricated utilizing thermoplastic vacuum-formed splints and, during the procedure, the printed teeth models served as templates for precise osteotomy and drilling. Once the replicas demonstrated a proper fit during the preparation of the recipient site, the extracted wisdom teeth were successfully transplanted.

**Figure 6:**
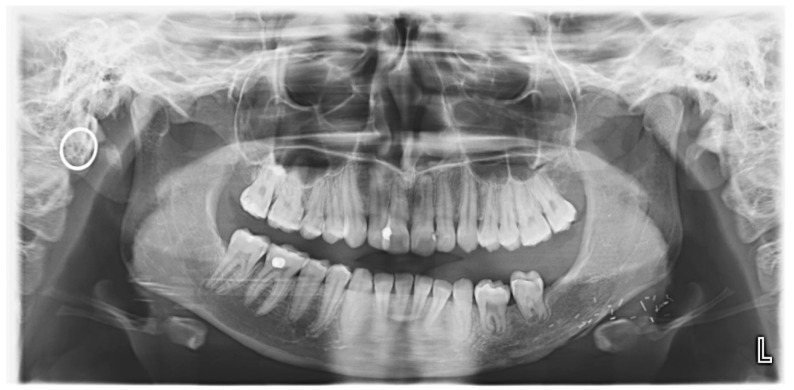
OPG at one-year follow-up after autotransplantation.

During the healing phase, the transplanted third molars exhibited persistent mobility, necessitating splint fixation for a total of twelve weeks. A wound dehiscence developed posterior to the tooth transplanted into region 37, leading to granuloma formation. Initially, a surgical resection of the granuloma was considered; however, spontaneous resolution was observed within twelve weeks of follow-up. Periodontal probing depths remained within normal limits, measuring less than 3 mm at the one-year follow-up. As expected, confirming pulp healing was challenging, as the transplanted teeth remained non-responsive to sensitivity testing. Consequently, endodontic treatment was not initiated, and a close follow-up routine was chosen to further monitor the transplanted teeth. Lastly, three months after the removal of the fixation, orthodontic treatment was initiated to achieve optimal tooth positioning (Figure 6).

At the one-year follow-up, quality of life was assessed using the validated Oral Health Impact Profile-49 (OHIP-49) and Harris Hip Score questionnaires. No reduction in head- and neck-related quality of life was observed. However, mild discomfort was reported at the donor site during heavy physical activity, resulting in good—but not excellent—hip function, with a score of 86 out of 100. Informed consent was obtained from the patient for the publication of this case report.

## 3. Discussion

This case reports the autotransplantation of two impacted third molars into a DCIA free flap in an adolescent patient. The procedure was performed six years after mandibular reconstruction, following the resection of an ossifying fibroma, ultimately resulting in successful complete oral rehabilitation.

This case is particularly significant due to the limited data available on this specific procedure. To our knowledge, no other reports have documented the use of 3D printing technologies for the autotransplantation of impacted wisdom teeth into a DCIA free flap in an adolescent patient. Despite DCIA flaps being the primary candidates for transplantation at this age, wisdom teeth remain an underutilized alternative to the conventionally used premolars. The integration of 3D technologies may further enhance the feasibility and precision of wisdom tooth autotransplantation, even in complex reconstructive cases.

The DCIA free flap is widely regarded as the optimal choice for preserving crestal height in young patients requiring complete dental rehabilitation. Although bony free flap surgery in adolescent patients remains uncommon, substantial evidence supports the autotransplantation of teeth into avascular bone grafts [13,14,15,16,17]. The existing literature suggests that periodontal recovery can be successfully achieved when periodontal ligament cells are transplanted along with the tooth into grafted bone [13].

Moreover, a medium-term follow-up case of tooth transplantation into an iliac crest free flap has demonstrated favorable success rates, further reinforcing the viability of this approach [18]. Tooth autotransplantation currently shows a survival rate comparable to that of dental implants [19,20,21]. Moreover, dental implants are generally not the preferred treatment option for adolescents due to the risk of positional changes, such as infraposition, which may lead to associated complications [3,6,7,8].

Wisdom teeth are frequently impacted, leading to potential complications over time. Consequently, extraction is often recommended for adolescents and young adults to prevent future oral health issues. However, they are not typically the first choice for autotransplantation due to several challenges, including anatomical variability, complex root morphology, and the technical difficulties associated with their extraction. These factors increase the risk of damage to the tooth during transplantation, compromising the procedure’s success [12,22].

Successful autotransplantation is generally more favorable when the donor tooth has incomplete root development, as this promotes revascularization and pulp healing [12,23,24]. Therefore, the optimal time for premolar transplantation is typically just before the age of twelve to fourteen years [20].

However, since wisdom teeth erupt late, their roots are often fully formed by the time they emerge, reducing the potential for successful integration [12,22,25,26].

In the case presented, the root development of the impacted wisdom teeth was incomplete at the time of transplantation. While we did not observe sensitivity due to the lack of sensory innervation of the DCIA free flap, we observed continued root growth. However, further assessment using radiographic follow-ups is needed to evaluate the revascularization of the transplanted teeth and possible indications for root canal treatment and apexification.

In cases where the transplanted teeth are not vital, to maintain a conservative approach given the young age of the patient, a valid alternative is represented by revitalization, with a success rate of 76.5% to 100% [27,28].

Advancements in 3D printing technology have helped address some of the limitations of autotransplantation in difficult cases. The use of 3D-printed surgical guides and replica models for preoperative planning enables a more precise positioning of the transplanted tooth, minimizing trauma to the periodontal ligament and enhancing overall outcomes [11,25]. As a result, the implementation of 3D imaging and printing techniques has been shown to reduce complication rates and enhance long-term treatment outcomes [11,13]. Given the generally high success rate of autotransplantation in grafted bone, it is worth exploring whether vascularized bony free flaps may provide even better outcomes than avascular bone grafts [13,14,15,16,17].

## 4. Conclusions

This case provides valuable insights into the possible medium-term success of third molar autotransplantation in reconstructive procedures. Our preliminary data from an adolescent patient undergoing non-malignant resection and reconstruction suggest a promising field of research for further investigations. Systematic research is needed to assess the role of tooth autotransplantation in bony free flaps. The integration of 3D printing technology could significantly enhance the feasibility of dental transplantation in challenging cases, particularly for suboptimal donor teeth such as wisdom teeth. By enabling precise surgical planning and optimizing recipient site preparation, this technology minimizes procedural challenges and improves overall outcomes. Additionally, 3D printing may help reduce damage to grafted teeth during transplantation, preserving their structural integrity and increasing the likelihood of successful engraftment. Additionally, research must evaluate its potential for optimizing long-term survival, functional rehabilitation, and broader application in more complex reconstructive cases.

## Data Availability

The data presented in this study are available solely on request from the corresponding author due to privacy and legal and ethical reasons.
